# Editorial: The role of dispersal and transmission in structuring microbial communities

**DOI:** 10.3389/fmicb.2022.1054498

**Published:** 2022-10-19

**Authors:** Kyle M. Meyer, Peter Deines, Zhong Wei, Posy E. Busby, Steven E. Lindow, Brendan J. M. Bohannan

**Affiliations:** ^1^Department of Integrative Biology, University of California, Berkeley, Berkeley, CA, United States; ^2^Zoological Institute, Christian Albrechts University Kiel, Kiel, Germany; ^3^College of Resources and Environmental Science, Nanjing Agricultural University, Nanjing, China; ^4^Department of Botany and Plant Pathology, Oregon State University, Corvallis, OR, United States; ^5^Department of Plant and Microbial Biology, University of California, Berkeley, Berkeley, CA, United States; ^6^Institute of Ecology and Evolution, University of Oregon, Eugene, OR, United States

**Keywords:** microbiome, transmission, dispersal, symbiosis, microbial community diversity

Microbial communities influence the systems they inhabit by driving ecosystem processes and promoting the health and fitness of plant and animals hosts. While an extensive body of work has documented variation in microbial community membership across hosts and systems, understanding the drivers of this variation remains a challenge. Much of the focus of these efforts has been on the characterization of host variation or the abiotic environment, and has overlooked the role of dispersal, i.e., the movement of organisms across space, and transmission, i.e., the movement of microbes among environments, hosts and between hosts and their environment. While quantifying or controlling the dispersal/transmission of microbial communities remains a technical and theoretical challenge, efforts to do so have led to insights into the assembly and function of microbial communities, highlighting the promising potential of a new wave of research. Our intention with this Research Topic is to draw attention to the important role that dispersal and transmission can play on the assembly, dynamics, and function of microbial communities and to highlight the unique ways that researchers are studying and conceptualizing microbial dispersal.

The study of dispersal and its implications for ecology and evolution have historically received more attention in macro-organismal communities (i.e., plants and animals) than microbial communities. Microbial ecologists can therefore take advantage of insights gained from plant and animal dispersal studies to examine similarities and idiosyncrasies among macro- and microorganisms. Custer et al. provide a foundational review of the topic of microbial dispersal and its implications for microbial ecology and evolution. This review also highlights where microbial and macro-organismal communities may differ in regards to their dispersal abilities, e.g., through spore formation or exceptionally long dormancy periods, and how microbial communities may exhibit forms of community-wide dispersal that are less common in macro-organismal communities, such as community coalescence.

One important avenue that plant and animal hosts acquire members of their microbiome is through vertical transmission from parent to offspring. While this form of transmission is known to occur, the mechanisms by which it takes place are rarely characterized, including whether one or both parents contribute members. Baldassarre et al. experimentally induce spawning in the estuarine sea anemone *Nematostella vectensis* to track maternal and paternal contributions to offspring microbiome assembly. They present evidence for distinct bacterial contributions from mothers and fathers that are consistent across replicate families, suggesting both parents play an important role in vertical transmission of microbiome members. In Bathia et al. researchers using the freshwater polyp *Hydra viridissima* demonstrate that the animal's vertically-transmitted algal photobiont *Chlorella* alters the outcome of microbiome assembly. By rearing aposymbiotic (algae free) polyps in co-culture with algae-containing polyps, they demonstrate that the animals assemble distinct microbiomes, suggesting a role of the vertical photobiont in microbiome assembly. Together these works suggest both direct and indirect effects of vertical transmission on the microbiomes of invertebrate animals.

While vertical transmission is one important route for host colonization, many microbiome members arrive through horizontal transmission, i.e., from other hosts or the environment. Bergmann and Leveau provide a comprehensive review of how both vertical and horizontal acquisition modes shape the plant microbiome. They suggest a re-framing of plant microbiome assembly through the lens of metacommunity theory, which integrates principles of ecological filtering, species interactions, and dispersal across multiple scales. Weinhold also suggests a re-framing of microbiome acquisition by taking into account the spatial extent of animal movement, e.g., territory size, foraging range, and clustering behavior. Here it is argued that differences in these behaviors will directly impact microbiome acquisition through differential exposure to microbial diversity, and indirectly impact acquisition by altering the effects of local environment on the host. In line with these suggestions, Tipton et al. tracked the dispersal of arbuscular mycorrhizal fungi (AMF) that were experimentally inoculated onto host plants. They demonstrate that non-inoculated nearby plant neighbors can act as host “bridges” thereby aiding the dispersal of AMF, which may help guide ecological restoration attempts. Lastly, Bongrand et al. demonstrate that the horizontally acquired Hawaiian bobtail squid (*Euprymna scolopes*) symbiont *Vibrio fischeri* can undergo significant genomic diversification during its residence in the animal host and thereby influence *V. fischeri* population genetic structure in the water column between transmission events to other *E. scolopes* hosts.

Microbial transmission dynamics may also influence the onset of host disease, either directly through pathogen transmission or indirectly through the transmission of microbial taxa that interact with pathogens. Using tall fescue grass, O'Keefe et al. tested whether *Epichloë coenophiala*, a vertically transmitted fungal endophyte, protects the host against the pathogen *Rhizoctonia solani*. Contrary to expectations, they demonstrate that experimental inoculation of *Epichloë* actually increased the prevalence of host disease despite its reported benefits on host growth. In a similar realm, Ishaq et al. review the literature on how interactions between microbial transmission and environmental change may impact the health, disease prevalence, and future population sizes of the American lobster (*Homarus americanus*). In this study, authors suggests that changes to the American lobster's habitat range due to ocean warming may relate to the recent increased incidence of epizootic shell disease, either through heightened stress of the host, or through increased exposure to the causative pathogen in the water column. Together, these two studies highlight a gap in our understanding of the interplay between pathogen transmission and microbiome assembly, suggesting that a closer examination of these two processes may help shed light on the variability of disease outcome, especially under changing environmental conditions.

Beyond host-associated microbial systems, the study of microbial dispersal can shed light on the variation of microbial communities across different environments. Hariharan and Buckley examine the spatial structuring and dispersal limitation of soil *Streptomyces* along two elevational gradients, one steep gradient (>1,000 m) and one relatively less steep (< 100 m). They conclude that the site with the steeper elevational gradient exhibits much higher beta diversity and suggest that this is likely driven by stronger species sorting due to sharper environmental changes. Maltz et al. also evaluate dispersal limitation using dust-associated microbial communities. Their study reveals effects of topography and elevation on microbial diversity and suggests that drought conditions may impose additional effects on dust microbial membership. Lastly, Hawkins and Zeglin examine how microbial inputs to the soil from bison dung may impact soil microbial community variation. They demonstrate that bison dung acts to increase local microbial diversity of the soil, but also drives increased community similarity over space, i.e. spatial homogenization. Taken together, these studies highlight that dispersal limitation and environmental selection are two crucial factors to better understand environmental microbial community assembly.

To summarize, these 12 papers highlight the importance of microbial dispersal and transmission for understanding microbial community assembly. Hosts have evolved numerous mechanisms to transmit members of their microbiome to their offspring and a better understanding of these mechanisms will undoubtedly help researchers make sense of the enormous variation that exists in microbiome membership. Insights gained from the spatial structuring of microbial communities both in hosts and the environment will also shed light on the diversity of microbial dispersal strategies and environmental preferences. Finally, as demonstrated by several contributions to this special issue, dispersal and transmission may have important implications for microbiome functionality, including disease progression, microbe-microbe interactions, or host health. Thus by bringing together this diverse body of work we hope to illustrate the multifaceted role that dispersal and transmission play in microbiome dynamics.

## Author contributions

KM wrote the editorial with input from all co-authors. All authors contributed to the article and approved the submitted version.

## Conflict of interest

The authors declare that the research was conducted in the absence of any commercial or financial relationships that could be construed as a potential conflict of interest.

## Publisher's note

All claims expressed in this article are solely those of the authors and do not necessarily represent those of their affiliated organizations, or those of the publisher, the editors and the reviewers. Any product that may be evaluated in this article, or claim that may be made by its manufacturer, is not guaranteed or endorsed by the publisher.

